# Field Application of a Rapid Spectrophotometric Method for Determination of Persulfate in Soil

**DOI:** 10.1371/journal.pone.0065106

**Published:** 2013-06-11

**Authors:** Colin J. Cunningham, Vanessa Pitschi, Peter Anderson, D. A. Barry, Colin Patterson, Tanya A. Peshkur

**Affiliations:** 1 Scottish Environmental Technology Network (SETN), Faculty of Engineering, University of Strathclyde, Glasgow, United Kingdom; 2 Laboratoire de technologie écologique, Institut des sciences et technologies de l'environnement, Faculté Environnement Naturel, Architectural et Construit (ENAC), École Polytechnique Fédéral de Lausanne (EPFL), Lausanne, Switzerland; Dowling College, United States of America

## Abstract

Remediation of hydrocarbon contaminated soils can be performed both *in situ* and *ex situ* using chemical oxidants such as sodium persulfate. Standard methods for quantifying persulfate require either centrifugation or prolonged settling times. An optimized soil extraction procedure was developed for persulfate involving simple water extraction using a modified disposable syringe. This allows considerable saving of time and removes the need for centrifugation. The extraction time was reduced to only 5 min compared to 15 min for the standard approach. A comparison of the two approaches demonstrated that each provides comparable results. Comparisons were made using high (93 g kg^−1^ soil) and low (9.3 g kg^−1^ soil) additions of sodium persulfate to a petroleum hydrocarbon-contaminated soil, as well as sand spiked with diesel. Recoveries of 95±1% and 96±10% were observed with the higher application rate in the contaminated soil and spiked sand, respectively. Corresponding recoveries of 86±5% and 117±19% were measured for the lower application rate. Results were obtained in only 25 min and the method is well suited to batch analyses. In addition, it is suitable for application in a small field laboratory or even a mobile, vehicle-based system, as it requires minimal equipment and reagents.

## Introduction

Chemical oxidation is most commonly applied as an *in situ* remediation technology but may also be applied *ex situ* to contaminated soils [1]–[3]. Common oxidants include persulfates, permanganates and peroxides [1]. Persulfate salts such as sodium persulfate (Na_2_S_2_O_8_) dissociate to the persulfate anion S_2_O_8_^−2^, which has a standard oxidation potential (E°) of 2.01 V. Activation by heat or ferrous iron generates a stronger oxidant in the form of sulfate radicals (SO_4_^−^•) with E° = 2.6 V.

It is advantageous to monitor the oxidant concentration at the time of application and during remediation to optimize the treatment process. Liang et al. [4] reviewed available methods for determination of persulfate in aqueous samples and presented a rapid spectrophotometric method that produced results after approximately 20 min. This represented a considerable time saving over previous techniques described by Shuiundu et al. [5] and Huang et al. [6]. The sodium persulfate CHEMets^®^ field test for aqueous samples (CHEMetrics Inc., Virginia, USA) uses the reaction between persulfate and ferrous thiocyanate to produce a color change that can be read using a comparator. The manufacturers highlight the possibility of interferences from hydrogen peroxide or ferrous ions, which are commonly used as activators of persulfate, as well as from cupric ions. Although the method is very rapid, the range is limited to a maximum concentration of 70 mg l^−1^, and like all comparator methods is subjective as it depends on the judgment of the operator. The addition of an efficient extraction step to the rapid spectrophotometric method of Liang et al. [4] would provide a valuable tool for assessing persulfate concentrations in soils.

Extraction of water-soluble ions such as persulfate from soils can be achieved using a variety of methods including ultrasound or mechanical shaking [4]. Ultrasound is unsuitable for this application as it enhances degradation of persulfate [7]. Mechanical shaking is a simple robust method of extraction that is easily deployed in a field laboratory. However, centrifugation of samples following extraction to separate the soil from the aqueous persulfate-containing solution adds considerably to the overall time and cost of determination.

*Ex situ* chemical oxidation treatments are often performed in slurry-based systems. Reducing the excess moisture content in the system to the point where chemical oxidation is still viable yields a soil that is more handleable. The authors are unaware of any previous work using sodium persulfate in non-slurry-based systems.

In this study, we describe the development of an optimized soil extraction procedure for persulfate that makes use of a modified disposable syringe for both extraction and separation of soil solids, thus removing the need for centrifugation. The use of syringes in soil testing has been described for soil sorption tests [8], [9] but have not been used for a combined field extraction and separation step as part of a spectrophotometric assay. The extraction time was optimized and potential interferences from extracts examined. A comparison between the modified syringe and centrifugation methods is presented using diesel-spiked sand and a petroleum hydrocarbon-contaminated soil from an industrial site.

## Materials and Methods

Experiments were designed to evaluate the efficacy of hydrocarbon removal using sodium persulfate. Two soil types were used. Clean sand was obtained from B&Q plc and a hydrocarbon-contaminated soil was sourced from an industrial site on the east coast of Scotland. Sodium persulfate was added at two concentrations, corresponding to high and low application rates as described below.

**Figure 1:**
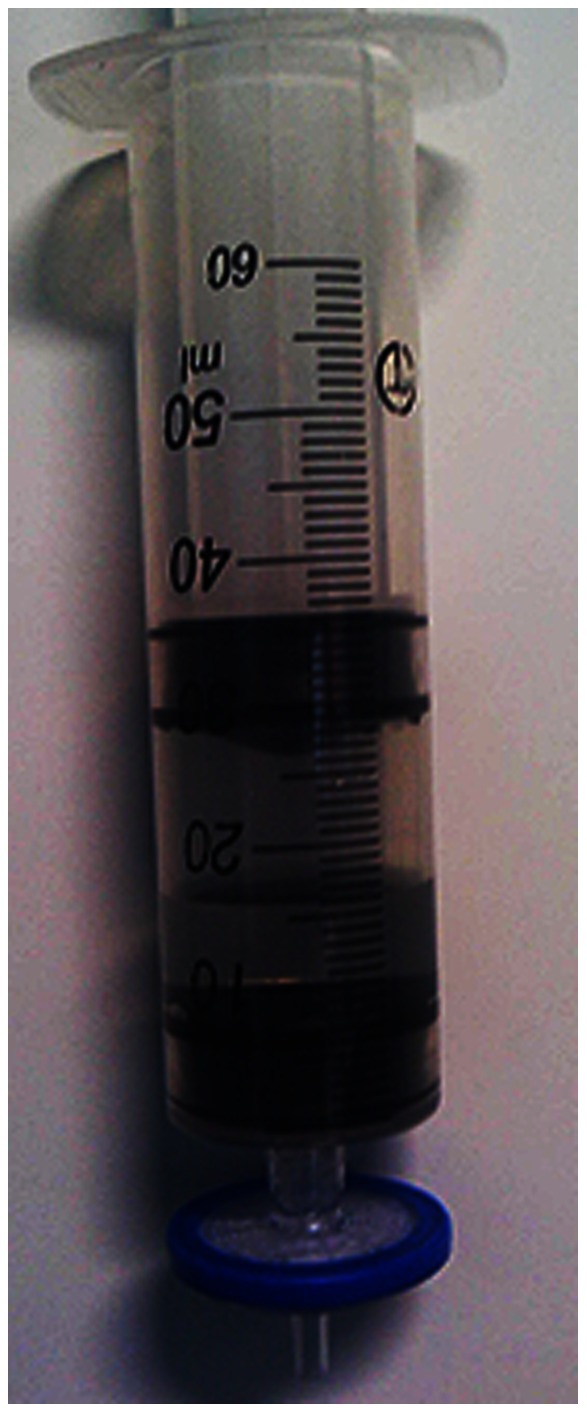
Photograph of the modified 50-ml syringe.

Analytical grade sodium persulfate (98%), potassium iodide (99.5%) and sodium hydrogen carbonate (99.9%) were purchased from Fisher Scientific Supplies. Absorbances were measured with a Jenway 6405 UV-Vis spectrophotometer using a 1-cm quartz cuvette. Disposable 50-ml Luer Lock tip syringes (Terumo) were used in the optimized method. These were modified by the addition of an inverted plunger from another syringe to the base of the barrel (Figure 1). The plunger had first been perforated 15 times with a sharp instrument, giving a cross-shaped perforation pattern, centered on the middle of the plunger. A pierced inverted plunger was used as a pre-filter to prevent blockages of the 0.45-µm nylon syringe filter (Fisher Scientific).

The contaminated soil and sand were sieved to 10 mm prior to use. The spiked sand was prepared by mixing 120 g of diesel into 6 kg of commercially cleaned sand in a black plastic bag to achieve a starting diesel concentration of 20,000 mg kg^−1^. The contaminated soil and spiked sand were characterized as follows. For all soil samples moisture was added to be compatible with on-site handleability. Moisture contents of the contaminated soil and spiked sand were determined to constant weight using a thermo-gravimetric technique (24 h at 105°C). Based on the measurement moisture contents, liquid phase concentrations were determined using the following equation:
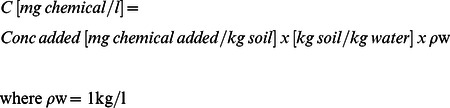



Total organic carbon (TOC) was determined using loss-on-ignition (LOI) at 550°C for 4 h in a muffle furnace and applying a conversion factor for soil by dividing by 1.8 [10]. Total petroleum hydrocarbons (TPH) were determined on samples extracted using accelerated solvent extraction (ASE 300, DIONEX) with a 1∶1 ratio of dichloromethane and acetone. Extracts were analyzed with a Thermo Focus gas chromatograph equipped with a flame ionization detector and Thermo AS 2000 auto-injector (Thermo-Fisher Scientific). Both the contaminated soil and sand were spiked with two concentrations of sodium persulfate powder (93.0 g kg^−1^ (620 g l^−1^) and 9.3 g kg^−1^ (62 g l^−1^)). Given that the solubility of persulfate in water is 730 g l^−1^ at 25°C [11] and the gravimetric moisture content of the contaminated soil was 15%, it was assumed that all the persulfate dissolved even at the highest application rate. The application rates of 620 g l^−1^ and 62 g l^−1^ equate to 85% and 8.5% of the maximum solubility limit for persulfate.

All soil and sand extractions were performed on triplicate 2-g samples with 20 ml of ultrapure water. In the standard method, extraction took place in 50-ml centrifuge tubes shaken for between 5 and 20 min on a wrist-action shaker (Stuart Scientific, Model SF1) then centrifuged at 2147 g for 20 min (ALC International Model PK130, Italy). In the syringe method, extraction took place in the modified syringes with the plunger in the barrel, leaving enough space for efficient mixing. The syringe was mixed using the wrist-action shaker for 5 min. All extracts were filtered through 0.45-µm syringe filters dispensing approximately 5 ml directly into 30-ml Sterilin containers.

Determination of persulfate concentration in the extracts followed the method of Liang et al. [1]. Briefly, a stock reagent solution containing 100 g l^−1^ of potassium iodide and 0.5 g l^−1^ of sodium hydrogen carbonate was prepared in ultrapure water. A 200-µl aliquot of each extract was added to 50 ml of the reagent solution in volumetric flasks. Closed flasks were inverted several times to mix then left to react for 15 min before determining the absorbance at 352 nm and 400 nm. The reagent solution was used as the blank. The total volume of 50.2 ml divided by 200 µl gave a dilution factor of 251.

**Figure 2:**
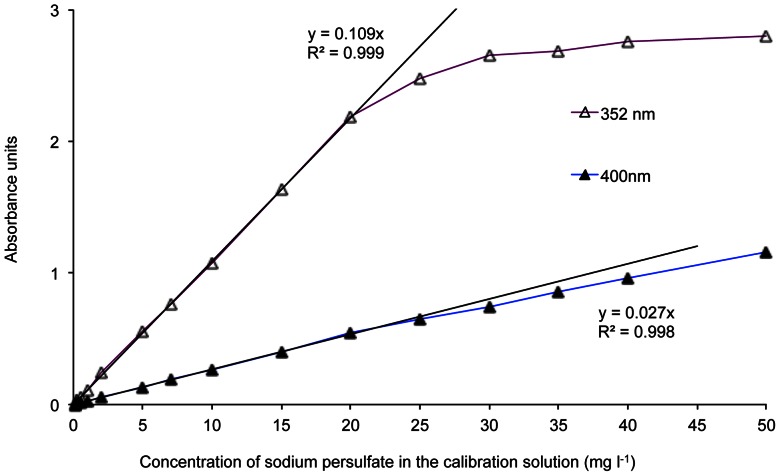
Calibration plot of absorbance with sodium persulfate solutions. Calibration plot of absorbance at 352 nm and 400 nm with solutions of standard sodium persulfate concentration from 0.1 to 50 mg l^−1^. The 352-nm plot is preferred for the low concentration measurements (down to 0.25 mg l^−1^) and the 400-nm plot for the high concentrations (up to 45 mg l^−1^).

**Figure 3:**
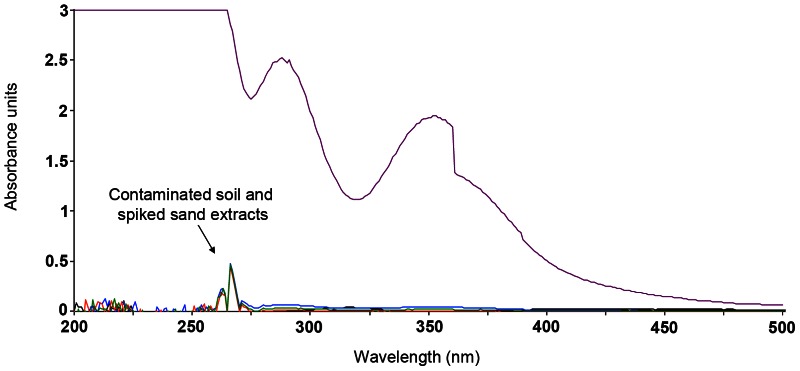
Absorbance spectra of potentially interfering elements present in the soil and sand matrix. Absorbance spectra of potentially interfering elements present in the soil and sand matrix compared to the absorbance spectra of an 18 mg l^−1^ sodium persulfate solution.

A calibration plot (Figure 2) was prepared for the 352 nm and 400 nm wavelengths with sample concentrations ranging between 0.01 mg l^−1^ and 50 mg l^−1^ sodium persulfate. To check for potential interferences, soil and spiked sand extracts were scanned between 200 and 500 nm (Figure 3). The detection limits for both wavelengths were determined by taking the standard deviation of 10 measurements of the reagent solution (blank) and multiplying this by a factor of three.

To establish the optimum shaking time, persulfate recovery was determined after 0, 5, 10, 15 and 20 min on both soil and spiked sand samples with the addition of 93.0 g kg^−1^ (620 g l^−1^) and 9.3 g kg^−1^ (62 g l^−1^) of sodium persulfate. These latter values were chosen, respectively, to represent a high initial application rate [12] and a low concentration where 90% of persulfate had degraded. A series of measurements were then made daily for a total of 4 d to test the ability of the method to monitor degradation of persulfate over time. All tests were conducted using the standard method.

The standard method using a centrifuge tube was then compared with the modified syringe method at the high and low concentrations on both contaminated soil and spiked sand on samples taken at 4, 5 and 6 d. Using Minitab 14, results were tested for normality. All data had a normal distribution and was therefore assessed using one-way analysis of variance (ANOVA). Significance is quoted at the 95% confidence level (p≤0.05). Finally, to further simplify the method and reduce the cost of each assay, some measurements were repeated where the 50-ml volumetric flasks were replaced by cheaper 30-ml Sterilins using 25 ml of reagent solution and 100 µl of extract.

## Results and Discussion

Contaminated soil had a LOI of 86.56 g kg^−1^ giving a TOC of 48.09 g kg^−1^. TPH was determined to be 6.67 g kg^−1^, pH was 7.7 and gravimetric moisture content was 15%. The total iron concentration was 25.97 g kg^−1^. The sand had a background LOI of 1.88, pH of 7.2 and a moisture content of 1%. Several days after spiking with diesel to 20.00 g kg^−1^ and immediately prior to the experiments, the TPH was determined to be 14.85 g kg^−1^. The total iron content in the sand was determined to be 15.12 g kg^−1^.

The calibration plots (Figure 2) showed that the persulfate assay was linear up to a concentration of 20 mg l^−1^ at 352 nm (*r* ^2^ = 0.999) and up to 45 mg l^−1^ (*r* ^2^ = 0.998) at 400 nm. Therefore, the 400-nm wavelength was required for higher persulfate concentrations such as those that would be found on site immediately after addition. The instrument detection limits were found to be 0.25 mg l^−1^ and 1.00 mg l^−1^, which equated to 0.63 g kg^−1^ and 2.51 g kg^−1^ in soil at 352 and 400 nm, respectively.

Liang et al. [4] selected the 352 nm and 400 nm wavelengths to avoid interference from iron compounds as these are commonly present in soils or added to activate persulfate prior to application. Scanning between 200 nm and 500 nm (Figure 3) showed there were no interferences at 352 nm or 400 nm wavelengths due to co-extracted materials from the contaminated soil or spiked sand samples. It is possible that other matrices could produce interferences and, as a precaution, it would be prudent to conduct a scan when working with new matrices.

**Figure 4:**
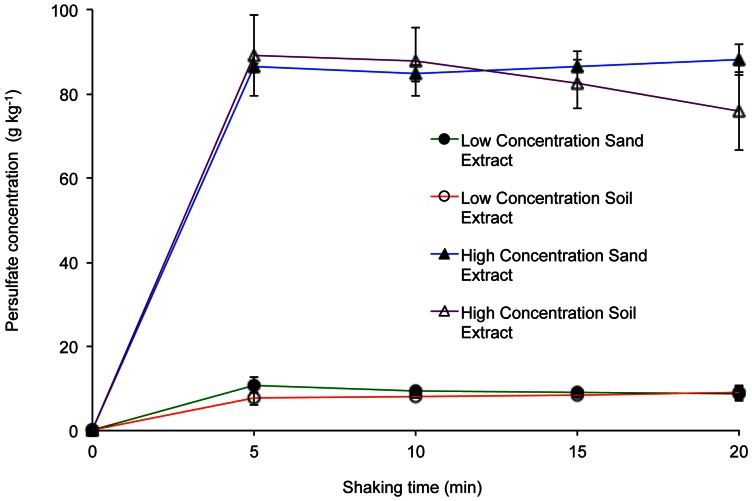
Soil and sand sodium persulfate recovery as a function of shaking time during extraction. Soil and sand sodium persulfate recovery as a function of shaking time during extraction. The top plots show the soil and sand spiked with an initial concentration of 93 g kg^−1^ of sodium persulfate. The lower plots are the results for soil and sand spiked with 9.3 g kg^−1^ of sodium persulfate. All tests gave a recovery greater than 90%. Error bars indicate the standard deviation (*n* = 3).

Examination of persulfate extraction efficiency with respect to shaking time (Figure 4) showed that 5 min was sufficient and provided good recovery in all cases. In the samples spiked with the higher 93 g kg^−1^ level of persulfate, contaminated soil produced a recovery of 88 g kg^−1^ (95±1%) and spiked sand a recovery of 89 g kg^−1^ (96±10%). At the lower persulfate addition rate of 9.3 g kg^−1^, recovery was 8 g kg^−1^ persulfate (86±5%) for the contaminated soil and 11 g kg^−1^ persulfate (117±19%) for the diesel-spiked sand.

**Figure 5:**
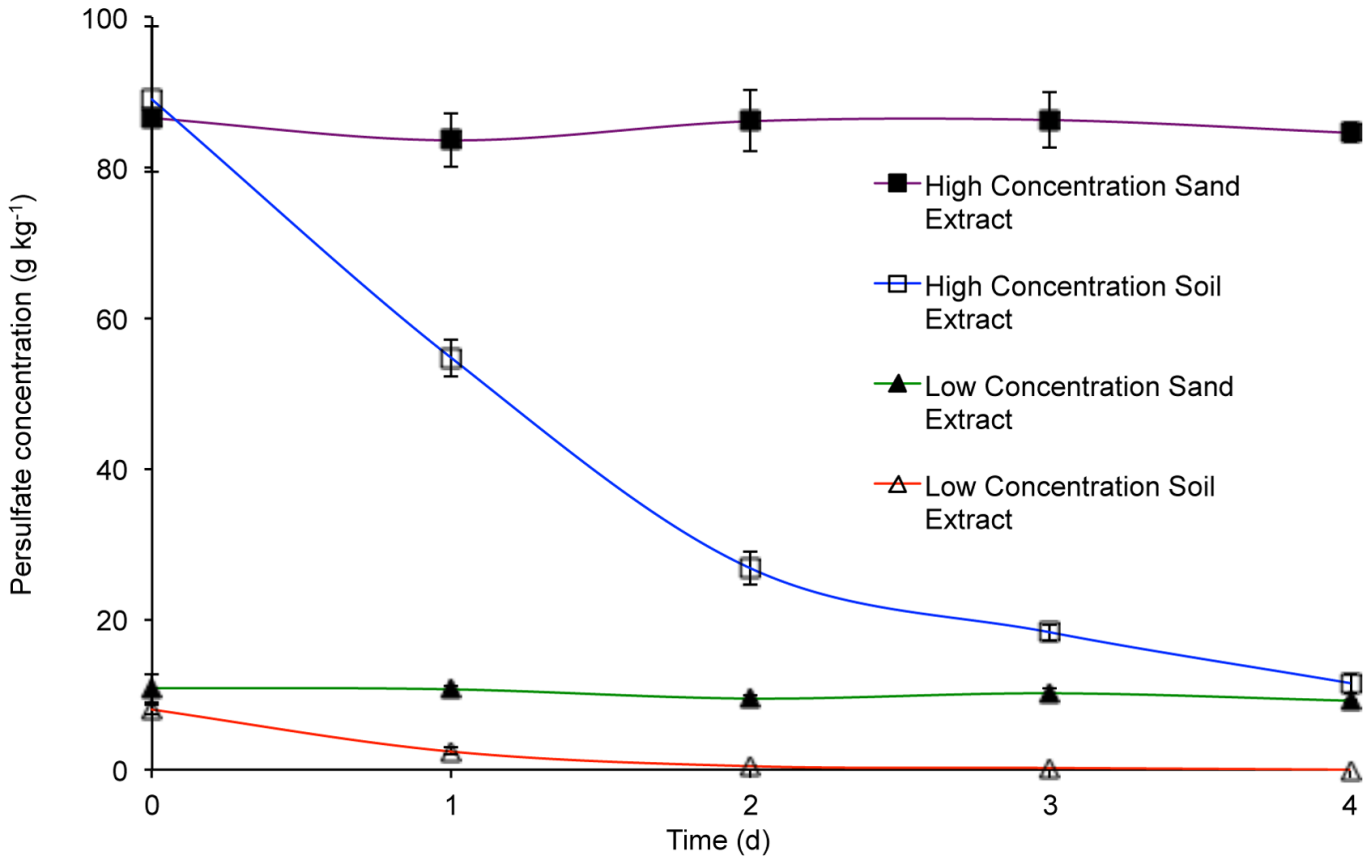
Natural persulfate degradation in contaminated soil and spiked sand with varying initial persulfate sodium concentrations. Natural degradation of persulfate in contaminated soil and spiked sand with high and low initial persulfate sodium concentrations. Initial concentrations were 93.0 g kg^−1^ for high level amendments and 9.3 g kg^−1^ for low level amendments. Error bars indicate the standard deviation (*n* = 3).

Figure 5 shows the sodium persulfate concentration over time in contaminated soil and spiked sand at high and low sodium persulfate addition rates. The rate of persulfate degradation observed in the spiked sand over time was generally low. This is explained by the low moisture content and relatively low background organic carbon content. Degradation of persulfate was easily monitored in the contaminated soil samples. At the lower (9.3 g kg^−1^) rate of application, persulfate was almost completely degraded after 2 d. Over the same period the greatest reduction was observed at the higher application rate (93 g kg^−1^), which continued to reduce by almost 90% to approximately 11.5 g kg^−1^ after 4 d.

**Figure 6:**
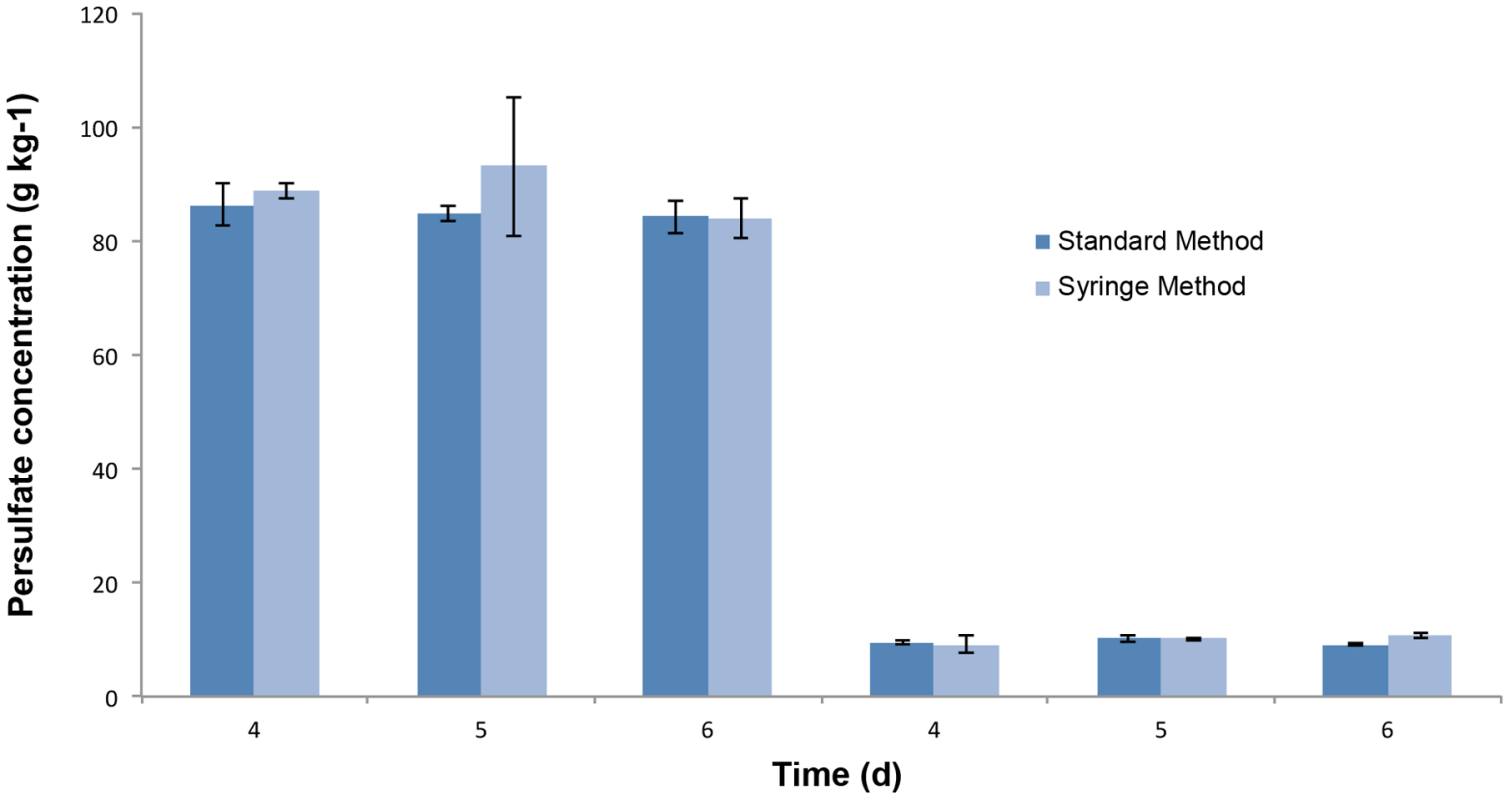
Results for sodium persulfate extraction of spiked sand with the standard method and modified syringe. Comparative results for sodium persulfate extraction with the standard method and the modified syringe method on spiked sand samples with high and low initial sodium persulfate concentrations. The extractions were carried out on days 4, 5 and 6 of the treatment. Error bars indicate the standard deviation (*n* = 3).

**Figure 7:**
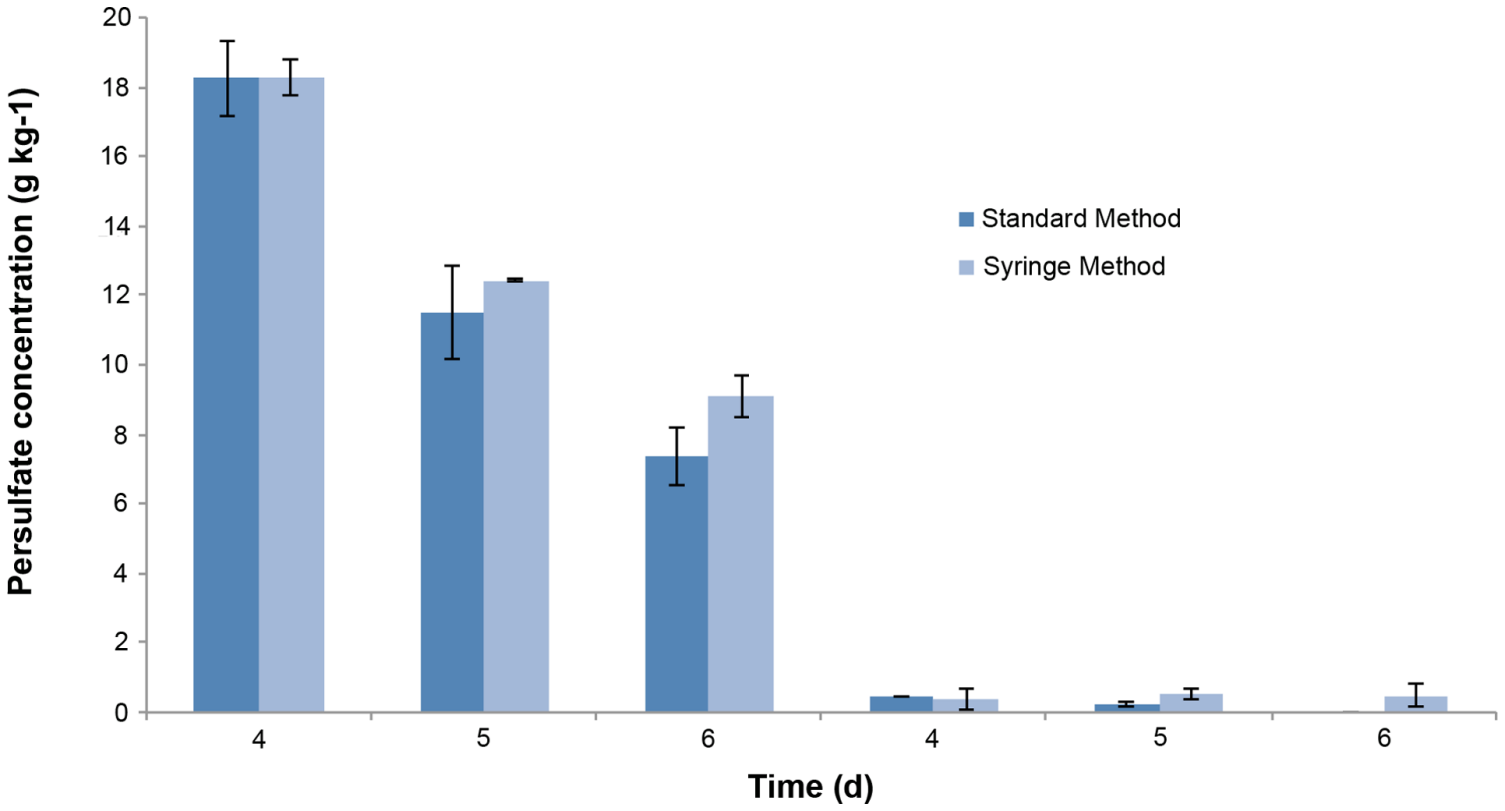
Results for sodium persulfate extraction of contaminated soil with the standard method and modified syringe. Comparative results for sodium persulfate extraction with the standard method and the modified syringe method on contaminated soil samples with high and low initial sodium persulfate concentrations. The extractions were carried out on days 4, 5 and 6 of the treatment. Error bars indicate the standard deviation (*n* = 3).

A comparison of the optimized syringe method versus the standard approach including a centrifugation step is shown for the spiked sand and contaminated soil in Figures 6 and 7, respectively. There were no significant differences between the standard method and the syringe method for the spiked sand extractions at both high and low persulfate concentrations with the exception of the high concentration extraction after 6 d where the syringe method gave a higher persulfate concentration. A similar trend was observed with the soil extractions. In this instance, there were no significant differences in the two extraction methods with the exception of the low concentration extraction where the syringe method had a higher persulfate concentration. These results indicate that the syringe method is comparable to the standard extraction method.

Liang et al. [4] reported that a limitation of the spectrophotometric assay they described was potential overestimation of persulfate around pH 2. In this study, all solutions assayed were between pH 4.5 and 6.8 with the exception of the highest addition rate of 93 mg g^−1^ sodium persulfate, which resulted in a pH of 2.0 in the contaminated soil. However, overestimation was not observed and the recovery of persulfate was found to be 95% (88 g kg^−1^).

## Conclusion

In this study, we described the development of a soil extraction procedure for persulfate for use in laboratory and field monitoring of *ex situ* chemical oxidation. A short extraction time of 5 min using a wrist-action shaker produced sufficient recoveries for determination by the rapid spectrophotometric method described by Liang et al. [4]. The extraction was optimized using a modified disposable syringe in place of extraction and centrifugation, with the new method yielding comparable results. Method detection limits of 0.63 g kg^−1^ or 2.51 g kg^−1^ were obtained depending on the wavelength (352 nm or 400 nm) used in the assay. The method is suitable for application in a small field laboratory. It could also be used as part of a mobile laboratory since it involves little equipment or reagents.
